# Endotoxemia is associated with an adverse metabolic profile

**DOI:** 10.1177/1753425920971702

**Published:** 2020-11-27

**Authors:** Anne-Mari Määttä, Aino Salminen, Milla Pietiäinen, Jaakko Leskelä, Teemu Palviainen, Wolfgang Sattler, Juha Sinisalo, Veikko Salomaa, Jaakko Kaprio, Pirkko J Pussinen

**Affiliations:** 1 Oral and Maxillofacial Diseases, University of Helsinki and Helsinki University Hospital, Helsinki, Finland; 2 Institute for Molecular Medicine Finland (FIMM), University of Helsinki, Helsinki, Finland; 3 Division of Molecular Biology and Biochemistry, Gottfried Schatz Research Center, Medical University of Graz, Graz, Austria; 4 Department of Cardiology, Heart and Lung Center, Helsinki University Central Hospital, Helsinki, Finland; 5 Department of Public Health Solutions, National Institute for Health and Welfare, Helsinki, Finland; 6 Department of Public Health, University of Helsinki, Helsinki, Finland

**Keywords:** Endotoxemia, infection, inflammation, lipoproteins, metabolic syndrome, metabolomics

## Abstract

Our aim was to analyze whether endotoxemia, i.e. translocation of LPS to circulation, is reflected in the serum metabolic profile in a general population and in participants with cardiometabolic disorders. We investigated three Finnish cohorts separately and in a meta-analysis (*n* = 7178), namely population-based FINRISK97, FinnTwin16 consisting of young adult twins, and Parogene, a random cohort of cardiac patients. Endotoxemia was determined as serum LPS activity and metabolome by an NMR platform. Potential effects of body mass index (BMI), smoking, metabolic syndrome (MetS), and coronary heart disease (CHD) status were considered. Endotoxemia was directly associated with concentrations of VLDL, IDL, LDL, and small HDL lipoproteins, VLDL particle diameter, total fatty acids (FA), glycoprotein acetyls (GlycA), aromatic and branched-chain amino acids, and Glc, and inversely associated with concentration of large HDL, diameters of LDL and HDL, as well as unsaturation degree of FAs. Some of these disadvantageous associations were significantly stronger in smokers and subjects with high BMI, but did not differ between participants with different CHD status. In participants with MetS, however, the associations of endotoxemia with FA parameters and GlycA were particularly strong. The metabolic profile in endotoxemia appears highly adverse, involving several inflammatory characters and risk factors for cardiometabolic disorders.

## Introduction

LPS, also known as endotoxin, is a major component of the outer membrane of most Gram-negative bacteria. Low to moderate LPS activity in serum most probably derives from microbiota colonizing the gastrointestinal tract, including the oral cavity. LPS originating from gut bacteria can enter the bloodstream either by direct diffusion due to intestinal paracellular permeability or by being aggregated into chylomicrons.^1^ Under physiological conditions, however, intestinal epithelium acts as a continuous barrier to avoid translocation of endotoxin, and LPS activity in serum remains low. A high-fat diet, obesity, diabetes, and non-alcoholic fatty liver disease have been associated with increased intestinal permeability, resulting in a two- to three-fold increase in levels of LPS in circulation.^2^ This condition, known as metabolic endotoxemia, associates with low-grade inflammation. In circulation, LPS is mainly carried by lipoproteins, most preferably high-density lipoproteins (HDL).^3^ HDL neutralizes LPS activity and clears LPS effectively from the bloodstream.^1^

Circulating LPS is involved in both acute infections and chronic conditions. It activates both innate and adaptive immune systems, leading to the production of Abs, cytokines and acute-phase proteins. Complete LPS is a complex lipoglycan composed of a core oligosaccharide, an O-specific side chain and a lipid A moiety, which, depending on its structure and the host, is responsible for the biological activity of the molecule. LPS is transferred from bacterial surfaces or LPS aggregates to the cell surface by LPS-binding protein, which is a positive acute-phase protein. The pro-inflammatory cascade mediated by NF-κB is triggered when LPS binds to the TLR4/CD14 complex expressed in almost all cell types.^4^ Via NF-κB, LPS also regulates metabolism by affecting gene transcription, e.g. to activate a signaling pathway leading to deteriorated insulin resistance.^4^

High LPS activity, i.e. endotoxemia, is linked to several cardiometabolic disorders and abnormalities. LPS activity correlates positively with serum total cholesterol as well as triglyceride concentrations and negatively with HDL cholesterol concentration.^5^ It is related to elevated C-reactive protein,^5^ insulin resistance and obesity.^6^ Moreover, endotoxemia associates with the risk of metabolic syndrome (MetS),^5,6^ predicts incident diabetes^5^ and is an independent risk factor for cardiovascular diseases (CVDs).^7,8^

We aimed to study whether endotoxemia, defined by the presence of LPS activity in the blood, associates with circulating metabolites including concentrations and compositions of lipoprotein particles, levels of fatty acids (FAs), Aas, ketone bodies, metabolic substrates, and other metabolic markers measured by NMR spectroscopy-based methods. Besides, we analyzed whether the associations are altered in cardiometabolic diseases, i.e. MetS and coronary heart disease (CHD).

## Materials and methods

### Participants

We investigated three Finnish cohorts: FINRISK97, FinnTwin16 and Parogene (total *n* = 7178). All the participants signed an informed written consent form. The study protocols were approved by the ethics committees of Helsinki University Central Hospital and Institute for Health and Welfare and were carried out according to the recommendations of the 1975 Declaration of Helsinki.

### The Finnish Twin Cohort Study (FinnTwin16)

FinnTwin16 is a longitudinal study consisting of 5563 Finnish mono- and dizygotic twins born in 1975–1979, and the twins were identified through the national population register of Finland. The study was established in 1991–1995 with baseline assessments of 16-yr-old twins and their parents. The follow-up questionnaires were conducted at ages 17, 18.5, and 23–27 yr.^9^ After the fourth assessment, a sample of twin pairs concordant and discordant for their alcohol use (*n* = 554 individuals) was selected for clinical examinations including the collection of blood samples.^10^ The participants were allowed to have a low-fat light breakfast, e.g. cup of coffee, bread or fruits, before the sampling.

### The Parogene Study

The original Corogene Study is based on 5295 Finnish patients who underwent coronary angiography for any reason at Helsinki University Central Hospital between June 2006 and March 2008. In addition to the angiogram, each patient filled out a questionnaire, and information was gathered from the medical records. The aim of the cohort study was to investigate genetic risk factors related to coronary artery disease.^11^ Approximately 10%, finally a total of 508 subjects, were randomly selected for a complete clinical oral examination which was performed 6 wk to 5 mo after the angiography. These patients constitute the Parogene Study.^12^ Of the Parogene Study patients, 123 (24.2%) had no significant coronary artery disease (< 50% stenosis), 184 (36.2%) had chronic coronary artery disease (≥ 50% stenosis), 169 (33.3%) had acute coronary syndrome (defined as a chest pain caused by myocardial ischemia and ≥ 50% stenosis), and 32 (6.3%) had acute coronary syndrome without coronary artery disease. Non-fasting blood samples were drawn from the patients.

### The FINRISK 1997 Study (FINRISK97)

The FINRISK 1997 Study is a prospective, random population-based survey aiming to monitor the health of the Finnish population. In the year 1997, 8444 individuals from five separate geographical regions in Finland were recruited to participate in clinical health examinations including questionnaires and blood sampling. The participants of the study were aged between 25 and 74. They were asked to fast for at least 4 h before blood sampling. The median fasting time was 5 h (interquartile range 3–7 h).^5^ Samples from a total of 6159 participants were available for the present analysis.

Some 1813 (29%) participants fulfilled the MetS diagnostic criteria of the International Diabetes Federation.^13^ Prevalent CVD covering myocardial infarction, coronary artery disease and stroke was defined as a self-report of doctor-diagnosed disease. Incident CVD events were recorded during the 13-yr follow-up time via national registers of hospitalizations, drug reimbursements and causes of death. There were 430 (7.0%) participants who had been diagnosed with prevalent CVD before the establishment of the study, and 649 (10.5%) participants were diagnosed during the follow-up. After excluding individuals who had experienced a stroke before the clinical survey in 1997 or during the follow-up, 365 (5.9%) and 395 (6.4%) participants remained in the groups of prevalent and incident CHD events, respectively.

### Serum metabolomics

All cohorts were analyzed with the same high-throughput serum NMR metabolomics platform as described previously in detail^14^ by Nightingale Health, Helsinki, Finland. There were data on 228 metabolites in FINRISK97 and Parogene and on 114 metabolites in FinnTwin16. The measures included concentrations and compositions of lipoprotein particles as well as FAs, Aas, ketone bodies, and other molecules involved in cell energy metabolism as well as ratios of lipoprotein lipid subclasses and FAs. We excluded some measures representing ratios of lipoprotein lipid subclasses in FINRISK97 and Parogene cohorts, leaving 157 metabolites for analyses. The 43 variables which best described the metabolite profile were selected for further analyses, including meta-analyses. Six classes of different sized very-low-density lipoprotein (VLDL) particles were re-grouped into two variables, large and small VLDL (XXL, XL and L VLDL as large VLDL; M, S and XS VLDL as small VLDL). Four subclasses of HDL were similarly re-grouped into two categories (XL and L HDL as large HDL; M and S HDL as small HDL). Our variables “branched-chain amino acids” and “aromatic amino acids” represent isoleucine, leucine and valine, as well as phenylalanine and tyrosine, respectively. Acetoacetate, acetate and β-hydroxybutyrate were combined as one variable denoting “β-oxidation.” Finally, these 43 metabolites comprised 11 lipoprotein, 11 lipid and eight FA-related measures, along with five Aas and eight molecules involved in cell energy metabolism. Measured metabolome data were available on 554 individuals in FinnTwin16, 6159 in FINRISK97 and 465 in the Parogene Study (7178 in total). FinnTwin16 lacked information on FA levels and consequently the meta-analysis in this respect was based only on the results of Parogene and FINRISK97 (*n* = 6624).

### LPS concentration measures

We analyzed serum LPS concentration by Limulus amebocyte lysate assay (HyCult biotechnology b.v., Uden, the Netherlands) with a chromogenic substrate according to the instructions provided by the manufacturer. The measurements were taken from distinct samples diluted (1:5 vol/vol) with endotoxin-free water. The inter-assay coefficients of variation were 7.4%, 9.2%^5^ and 5.5%^15^ for FinnTwin16, FINRISK97 and Parogene samples, respectively.

### Statistical analyses

R (version 3.6.1 or higher, http://www.r-project.org/) was used for all statistical analyses.

The LPS activity and metabolic measures in FINRISK97 with highly skewed distribution (Pearson’s moment coefficient of skewness < –1.5 or > 1.5) were log-transformed to obtain approximately normal distributions. Subsequently, concentrations were scaled to a mean of zero and a standard deviation of one. The transformations were performed similarly for the corresponding metabolites in Parogene and FinnTwin16. Generalized linear regression models were fitted between LPS activity as an outcome and metabolite concentrations as predictors for Parogene and FINRISK97 separately. For FinnTwin16, a linear mixed-effects model was fitted to take account sample relatedness using family number as the random effect of the model. The models were adjusted for age, sex, body mass index (BMI), and current smoking status (smoker or non-smoker). To combine the results from individual cohorts, an inverse-variance weighted fixed-effect meta-analysis was performed.

FINRISK97 was further analyzed after grouping participants according to MetS, BMI, CHD status and smoking habit. Linear regressions were calculated for these groups separately, and to assess possible differences, we extended the main model by interaction terms of the grouping variables and conducted ANOVA tests. The statistical tests were two-sided. In addition, we acquired predictors from logistic regression of MetS and covariates including age, sex, smoking, weekly alcohol intake and years of education. Then, logistic regression models were fitted between the predictors and five metabolites of interest with and without LPS. To illustrate the results, receiver-operating characteristics (ROC) curves were drawn.

Because there were strong correlations among metabolites, a principal component (PC) analysis was conducted for 157 metabolic measures of FINRISK97. Metabolites with factor loadings ≤ –0.10 or ≥ 0.10 were considered to contribute substantially to the PC. General linear regression models adjusted for age, sex, BMI and current smoking were calculated between LPS and scores of the 22 first PCs.

## Results

We analyzed the associations between LPS and circulating metabolites in three Finnish cohorts comprising altogether 7178 individuals. The characteristics of the cohorts are summarized in Table 1. The FINRISK97 study is population based (49% women and a mean age of 50 yr), Parogene consists of patients who underwent coronary angiography (31% women and a mean age of 64 yr) and FinnTwin16 is composed of young adult twins (49% women and a mean age of 26 yr).

**Table 1.** Characteristics of the study cohorts.

	FINRISK97	Parogene	FinnTwin16
Study design	Population-based cohort	Random series of patients referred for coronary angiography	Young adult twins
*n*	6,159	465	554
Women (%)	3,027 (49.1)	163 (35.1)	273 (49.3)
Mean age, yr	53.0 (10.6)	63.6 (9.0)	26.2 (1.4)
Current smokers (%)	1,376 (22.3 )	55 (11.8)	229 (41.3)
Mean BMI, kg/m^2^	27.2 (4.5)	27.8 (5.0)	23.9 (4.0)
Underweight, <18.50 (%)	29 (0.5)	5 (1.1)	12 (2.2)
Normal weight, 18.50–24.99 (%)	2,025 (32.9)	134 (28.8)	351 (63.4)
Overweight, 25.00–29.99 (%)	2,718 (44.1)	200 (43.0)	128 (23.1)
Obese, ≥ 30.00 (%)	1,376 (22.3)	122 (26.2)	34 (6.14)
Diabetes mellitus (%)	438 (7.1)	108 (23.2)	0 (0)
Lipid-lowering medication (%)	269 (4.4)	370 (79.6)	0 (0)
Mean LPS concentration, EU/ml	0.63 (0.37)	0.63 (0.44)	0.67 (0.23)
Total cholesterol, mmol/l*	5.47 (1.07)	3.28 (0.82)	4.98 (0.89)
LDL cholesterol, mmol/l*	2.00 (0.58)	1.10 (0.44)	1.87 (0.52)
HDL cholesterol, mmol/l*	1.57 (0.39)	1.20 (0.33)	1.77 (0.42)
Total triglycerides, mmol/l*	1.38 (0.70)	1.25 (0.62)	1.30 (0.60)

Numbers indicate *n* (%) or mean (standard deviation) when appropriate. *Concentrations acquired by NMR.

We conducted a PC analysis for 157 metabolites measured from FINRISK97. The first 22 PCs explained more than 95% of the variation of the data (Supplemental Table 1). Thus, the *P* value threshold for statistical significance was set to 0.05/22 = 0.0023 in further analyses.

**Figure 1.**
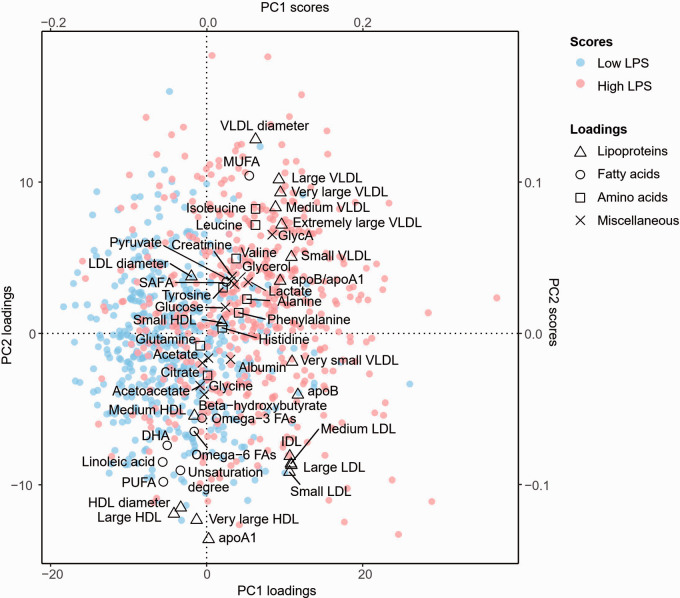
Factor loadings and scores of the first two principal components in FINRISK97. The loadings of the designated metabolites are shown. FAs represent their ratios to total FA concentration. The figure also illustrates the scores of subjects belonging to the highest and lowest deciles by their LPS values.

**Figure 2.**
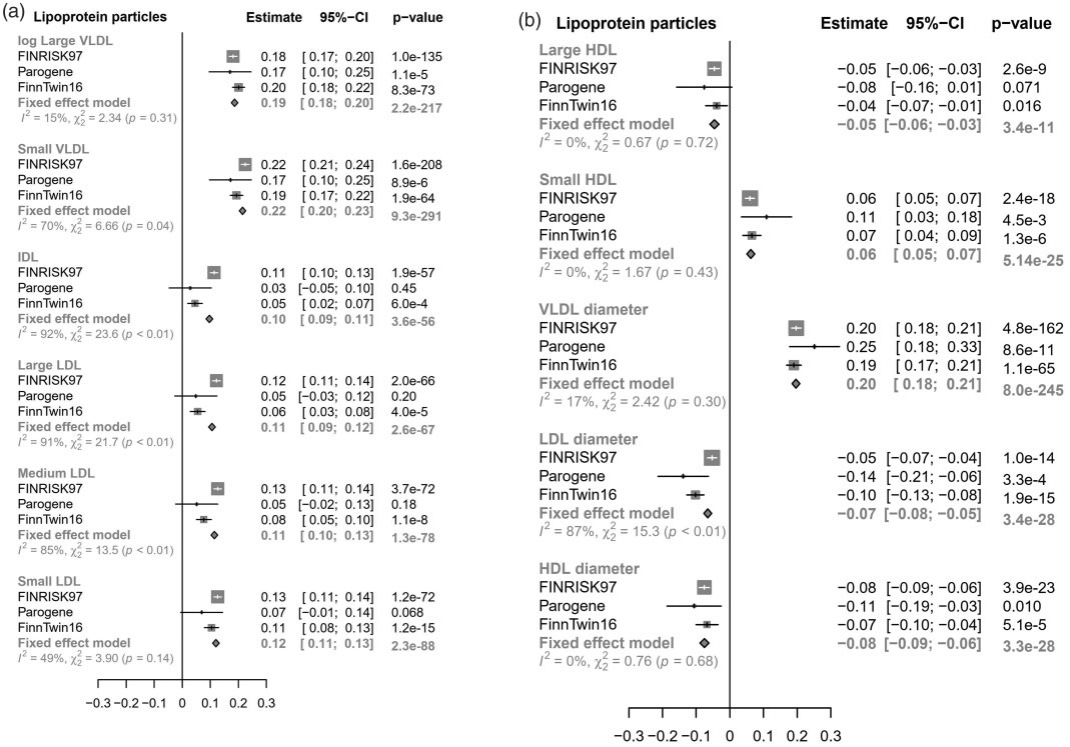
The associations of endotoxemia with (a) concentrations of VLDL, IDL and LDL, and (b) concentrations of HDL particles and diameters of lipoprotein particles. The β-values and 95% confidence intervals from the analyses of the individual cohorts and from inverse-variance weighted fixed-effect meta-analysis are presented. The linear regression models were adjusted for age, sex, BMI, and current smoking status, as well as kinship in FinnTwin16. Metabolites labeled with “log” were log-transformed to obtain normal distributions.

**Figure 3.**
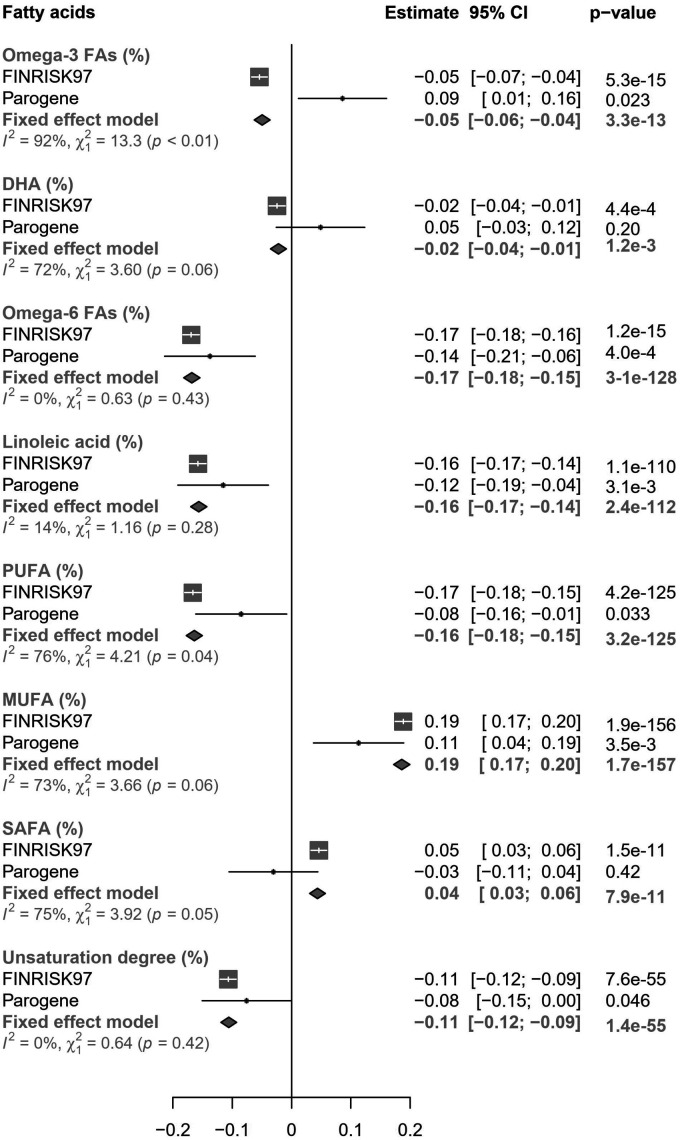
The associations of endotoxemia with fatty acids. The β-values and 95% confidence intervals from the analyses of the individual cohorts and from inverse-variance weighted fixed-effect meta-analysis are presented. The linear regression models were adjusted for age, sex, BMI, and current smoking status.

We included altogether 157 serum metabolites in our linear regression models of the separate cohorts and 43 metabolites in the meta-analysis combining the results. The mean values and standard deviations of the metabolite measures in the different study cohorts are presented in Supplemental Table 2. Supplemental Figure 1 shows LPS distributions in each cohort. According to the meta-analysis, all 43 metabolites were significantly associated with endotoxemia (*P* < 0.0023), and these results are shown in Figures 1–3 and Supplemental Figure 2.

### Principal component analysis

We studied the associations between endotoxemia and the first 22 PCs acquired from the metabolite measures in FINRISK97 (Supplemental Table 1). The first three PCs associated directly with endotoxemia. PC1 had positive factor loadings of VLDL parameters, apoB, and FAs including monounsaturated (MUFA) and saturated (SAFA) fatty acids (Figure 1), whereas PC2 was characterized by a large mean diameter of VLDL, low HDL and low-density lipoprotein (LDL) cholesterol contents, in addition to small HDL particle size. PC3 comprised high levels of lipid-rich HDL particles and FAs together with MUFA and SAFA, as well as a low ratio of apolipoprotein B (apoB) to apolipoprotein A1 (apoA1).

### Lipoprotein associations with endotoxemia

The associations of LPS activity with concentrations and mean diameters of lipoprotein particles are shown in Figures 2a and 2b. Endotoxemia was positively associated with concentrations of chylomicrons and VLDL, as well as intermediate-density lipoprotein (IDL) and LDL particles in the meta-analysis. Endotoxemia was inversely associated with the concentrations of large HDL particles, whereas there was a direct association with concentration of small HDL particles. A weak association was also observed between endotoxemia and total HDL concentration in FINRISK97 (β = 0.02, 95% confidence interval (CI) 0.01, 0.04, *P* = 0.0006), but in other cohorts or in the meta-analysis this association was not significant (*P* = 0.007). Among the particle sizes, endotoxemia was directly associated with VLDL diameter, while the associations with the diameters of LDL and HDL were inverse.

The associations between endotoxemia and apolipoproteins are presented in Supplemental Figure 2. A small but statistically significant association with endotoxemia was observed for apoA1, whereas the associations for apoB and apoB/apoA1-ratio were considerably stronger. The figure also illustrates the associations between endotoxemia and total lipids in lipoprotein particles. Positive associations were found with total lipids of VLDL and IDL, as well as LDL. Among the total lipids in HDL particles, there was an inverse association with large HDL, and a direct association with small HDL particles. The associations of endotoxemia with all the measured metabolites including exact lipoprotein lipid subclasses in separate cohorts are presented in Supplemental Table 3.

### Endotoxemia and fatty acids

Total FAs and all the other measured absolute levels of FAs associated directly with endotoxemia (Supplemental Table 2). However, as shown in Figure 3, among relative concentrations of FAs, we observed more heterogeneity: unsaturation degree of FAs, proportions of polyunsaturated fatty acids (PUFA), including omega-3 FAs, docosahexaenoic acid (DHA), and omega-6 FAs, including linoleic acid were inversely associated with endotoxemia, whereas endotoxemia was directly associated with proportions of MUFA and SAFA.

### Other molecules

**Figure 4.**
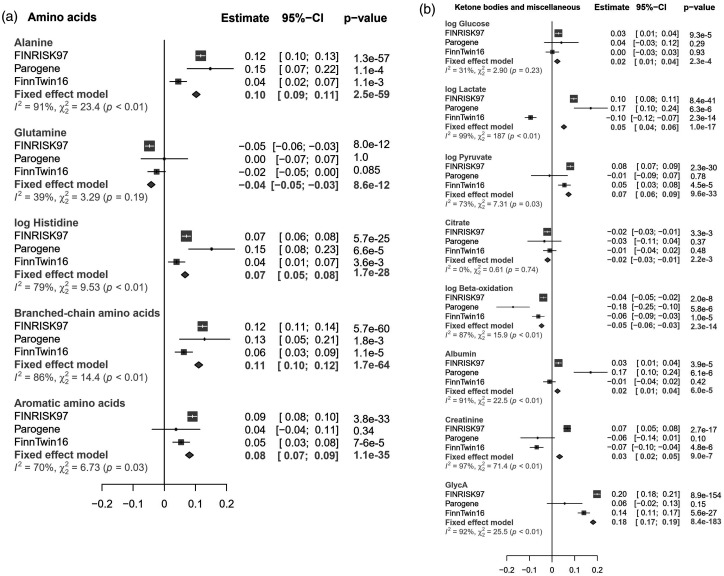
The associations of endotoxemia with (a) Aas, and (b) metabolic substrates, ketone bodies and other metabolic markers. The β-values and 95% confidence intervals from the analyses of the individual cohorts and from inverse-variance weighted fixed-effect meta-analysis are presented. The linear regression models were adjusted for age, sex, BMI, and current smoking status. Metabolites labeled with “log” were log-transformed to obtain normal distributions.

Aromatic Aas, phenylalanine and tyrosine, were positively associated with endotoxemia (Figures 4a and 4b). Among Aas, however, the strongest associations were found for branched-chain Aas, isoleucine, leucine and valine. In addition, we observed positive associations of endotoxemia with alanine and histidine, and inverse association with glutamine. Among glycolysis- and gluconeogenesis-related metabolites, Glc was positively associated with endotoxemia, along with lactate, pyruvate and glycerol, while only citrate was negatively associated with LPS. Acetoacetate, acetate and β-hydroxybutyrate originating from β-oxidation were directly associated with endotoxemia. There were also positive associations for albumin, creatinine, and GlycA.

### Endotoxemia and MetS

**Figure 5.**
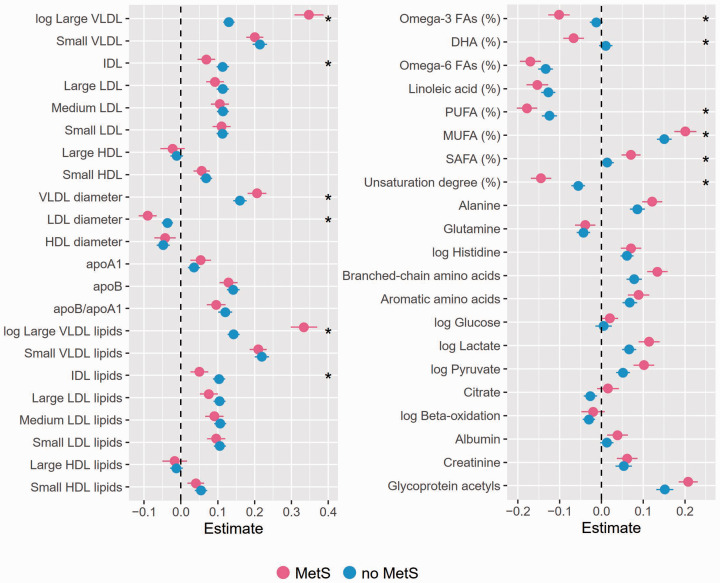
The associations between LPS and metabolites in subjects with and without metabolic syndrome (MetS) in FINRISK97. The subjects of FINRISK97 were grouped according to the MetS status. General linear regression models adjusted for age, sex, BMI, and current smoking status were fitted between LPS and the metabolites in each group. The significance of the differences in the regressions between the groups was analyzed by adding an interaction term of the MetS status with metabolite concentration in the main model and conducting ANOVA. The β-values and 95% confidence intervals are presented. Metabolites labeled with “log” were log-transformed to obtain normal distributions. *: *P* < 0.0023 (ANOVA).

**Figure 6.**
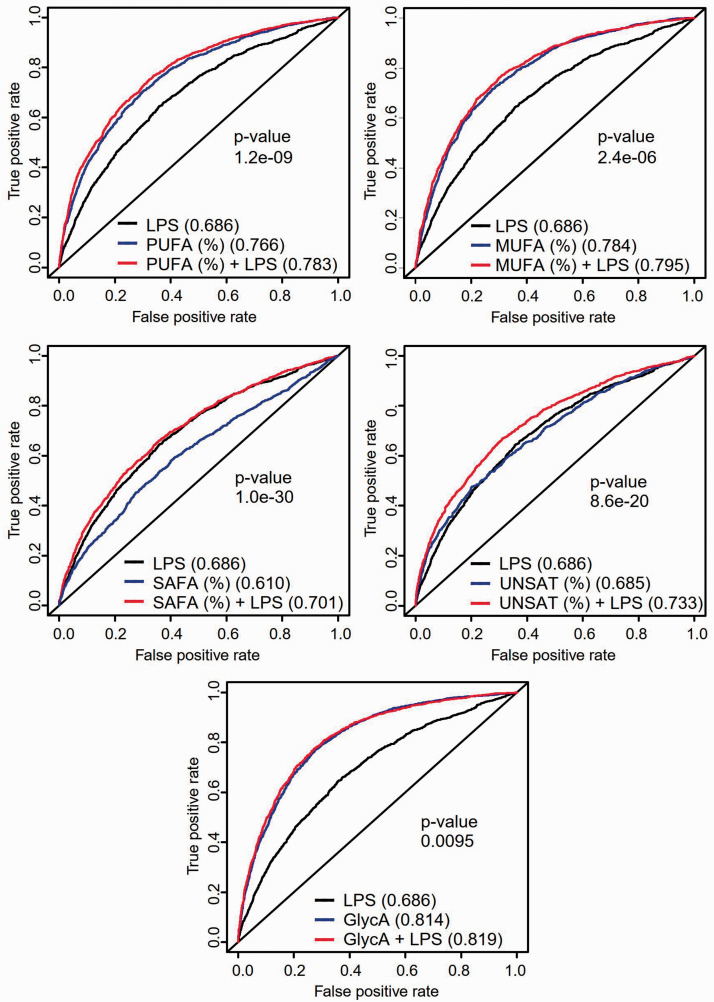
ROC curves with MetS as a response and metabolites and LPS as predictors in FINRISK97. We acquired the predictors from logistic regression of MetS and covariates including age, sex, smoking, weekly alcohol intake and years of education. Then, logistic regression models were fitted between the predictors and the metabolites of interest with and without LPS. AUC-values for metabolites are presented. P-values depict the discrimination ability of the models with and without LPS. PUFA, polyunsaturated fatty acids; MUFA, monounsaturated fatty acids; SAFA, saturated fatty acids; UNSAT, unsaturation degree of fatty acids; GlycA, glycoprotein acetyls.

Next, we analyzed whether the associations between endotoxemia and the metabolites differ between subjects with and without MetS in FINRISK97 (Figure 5). We detected a significantly stronger positive association of endotoxemia with concentrations and total lipid contents of large VLDL and IDL, as well as VLDL diameter among subjects with MetS compared with those without. Similarly, the participants with MetS had a significantly stronger positive association between endotoxemia and relative levels of omega-3 FAs, DHA, PUFA, MUFA, and SAFA, as well as unsaturation degree of FAs. The effect of endotoxemia on the relationships between MetS and five metabolites of interest was analyzed further. LPS (OR, 95% CI; 2.00, 1.87–2.13), MUFA (3.26, 3.02–3.53), PUFA (0.33, 0.31–0.35), SAFA (1.47, 1.39–1.56), unsaturation degree (0.48, 0.45–0.51) and GlycA (3.82, 3.52–4.15) were all strongly associated with MetS. The discrimination ability of the models with and without LPS were compared with C-statistics, and the area under curve (AUC) values (Supplemental Table 4) and ROC curves are presented (Figure 6). LPS alone provided an AUC of 0.686 (0.671–0.701) to differentiate participants with and without MetS. Including any of the five metabolites in the model increased its discrimination ability significantly (Supplemental Table 4). Adding LPS in the models with unsaturation degree of FAs or proportion of SAFA clearly improved the AUCs. The best discrimination, however, was achieved with LPS and GlycA providing an AUC of 0.819 (0.807–0.831).

### Endotoxemia and CHD status, smoking and BMI

We also analyzed whether the associations between endotoxemia and the metabolites differ between subjects with varying CHD status, current smokers and non-smokers, and BMI categories. These results are presented in Supplemental Figures 3–5.

The CHD status, i.e. no CHD, prevalent CHD, and incident CHD, affected significantly only the associations of endotoxemia with concentrations of large VLDL particles and their total lipids. Compared with non-smokers, the current smokers presented a stronger association of endotoxemia with LDL diameter, proportion of linoleic acid, and glycerol. Among BMI categories, i.e. underweight or normal weight, overweight, and obese, significant differences were found in associations of endotoxemia with large VLDL particles and their lipid content, IDL lipid content, albumin, and omega-3 FAs.

## Discussion

In our large study conducted among over 7000 participants, we showed that endotoxemia is associated with high concentrations of VLDL, IDL and LDL particles, and low concentrations of HDL particles. It is also associated with large VLDL, but small LDL and HDL particle diameters. In addition, there was a direct association between endotoxemia and FAs with a high saturation degree. Also notable was the positive association of endotoxemia with aromatic and branched-chain Aas, as well as GlycA, and Glc. Some of these disadvantageous associations were significantly stronger in smokers and subjects with high BMI, but especially in participants with MetS. The metabolic profile in endotoxemia appears highly adverse, involving several inflammatory characters and risk factors for cardiometabolic disorders.

The process of lipoprotein remodeling is a major manifestation of aberrant metabolic pathway utilization occurring in inflammatory diseases.^16^ These changes in lipoprotein composition affect both the host immune response (due to different LPS inactivation efficacy of different lipoprotein classes) and the host metabolic phenotype. In terms of metabolism, this is substantiated by findings that LPS induces adipose tissue lipolysis, providing an increased supply of free FAs that are subject to hepatic uptake. In the liver, decreased β-oxidation together with increased FA synthesis results in increased hepatic *de novo* lipogenesis. Increased VLDL synthesis and secretion is observed as the net effect of overshooting hepatic triglyceride synthesis.^17^ A decrease in lipoprotein lipase activity leads to delayed clearance and prolonged circulation of triglyceride-rich lipoproteins during inflammatory conditions. In line, the present study revealed a positive association of metabolic endotoxemia with concentrations, total lipid content, and mean diameter of VLDL particles.

Krauss described two distinct LDL phenotypes, (i) the predominance of large buoyant (lb)LDL particles (phenotype A) and (ii) conditions where small dense (sd)LDL particles (phenotype B) predominate.^18^ Results obtained during the present study indicated the preponderance of sdLDL particles in metabolic endotoxemia. Whether sdLDL are produced from triglyceride-rich VLDL or lbLDL is not entirely clear and depends on the underlying metabolic condition.^19^ These sdLDL particles have a low affinity to the LDL receptor, enter the arterial wall and are highly susceptible to oxidative modifications leading to enhanced uptake by macrophages,^20^ a process ultimately giving rise to subendothelial foam cell formation.

Peripheral inflammation also impacts HDL structure and function through alterations in the lipid and protein moieties. These changes affect anti-inflammatory, anti-oxidative and reverse cholesterol transport properties of HDL particles.^21^ Here, we found a negative association between endotoxemia and the mean diameter of HDL particles. There was a slightly positive association for apoA1, the most prominent protein in HDL, but no association between endotoxemia and total HDL concentration. Thus, our findings concerning HDL do not exactly meet the characteristics of acute-phase HDL. During the acute-phase response, the concentration of HDL decreases, while the particle undergoes phospholipid transfer protein-mediated conversion,^22^ and apoA1 is replaced by serum amyloid A.^23^ These alterations are seen in inflammatory diseases such as rheumatoid arthritis, MetS and atherosclerosis, as well as infections.^20^ Many of these diseases are also associated with a higher risk for CVD.^20^ Large and medium-sized HDL are more effective in reverse cholesterol transport than small HDL.^24^ In our study, high endotoxemia was associated with concentrations of small HDL, whereas large HDL particles associated with lower endotoxemia. It is reasonable to assume that this dysfunctional HDL profile probably predicts poor cholesterol efflux capacity.

Diabetic dyslipidemia is a condition interrelated with insulin resistance and type 2 diabetes mellitus (T2DM). This disorder is characterized by hepatic overproduction of large, triglyceride-rich VLDL.^25^ Highly atherogenic sdLDL is another feature of lipoprotein profile observed in diabetic dyslipidemia.^25^ These alterations in the lipoprotein profile of subjects with MetS may explain the differences we observed here between the groups. We showed that endotoxemia associated more strongly with concentrations and lipid contents of the larger VLDL population among participants with MetS compared with those without. VLDL diameter was also more strongly associated with endotoxemia in MetS. In addition, in this group, we found that the negative association between endotoxemia and LDL diameter was more remarkable, emphasizing the abundance of small, dense LDL in MetS.^26^

As mentioned above, changes in the compositions and concentrations of plasma lipids affect the host immune response due to LPS binding by lipoproteins. Lipoproteins are generally considered as protective during LPS-mediated inflammation, as the majority of LPS is bound to circulating lipoproteins.^3^ HDL appears to inactivate LPS with highest efficacy, whereas lipoproteins with lower densities are less efficient in neutralizing its function.^27^ In contrast to physiological conditions, LDL or VLDL particles are the major LPS carriers in patients with sepsis.^27^ LPS redistribution between different lipoprotein classes is dependent on LPS-binding protein and phospholipid transfer protein,^28^ a lipid transfer enzyme and major effector of lipoprotein particle size.^22^ Of note, we have previously demonstrated that the specific activity of phospholipid transfer protein is significantly up-regulated in severe acute-phase response patients.^22^ As we observed a positive relationship between endotoxemia and small HDL diameter but no association for total HDL concentration, our findings suggest that HDL subclasses of different sizes vary in their capability of neutralizing LPS.

Findings obtained during the present serum NMR metabolomics study indicate substantial association between endotoxemia and a number of metabolites frequently associated with MetS or T2DM.^29^ Endotoxemia can induce insulin resistance that is, among other features, characterized by reduced Glc utilization in adipose tissue and skeletal muscle, resulting in increased adipose tissue lipolysis, skeletal muscle proteolysis, and hepatic gluconeogenesis to compensate for energy deficits.

During the present study, we observed a positive association of endotoxemia with concentrations and several markers of FA composition and saturation in both the FINRISK97 and Parogene cohorts. These findings would be compatible with increased adipose tissue lipolysis resulting from limited GLUT4-mediated Glc availability as observed in MetS and insulin resistance. When the subjects of the FINRISK97 study were grouped according to the MetS status, PUFA, omega-3 and DHA levels (all negatively associated with endotoxemia) were significantly different in the MetS group. In contrast, the positive associations with MUFA and SAFA were significantly higher in the MetS population. Whether this is a result of aberrant redox control experienced by patients with MetS^30^ and thus consumption of highly unsaturated FAs due to higher oxidative stress conditions, remains to be elucidated. Also, the reasons for the negative association with ketone bodies acetate, acetoacetate and β-hydroxybutyrate, which are synthesized from FA-derived acetyl-coenzyme A, are currently unclear.

In the Aa cluster, several metabolites considered as biomarkers in the setting of poor glycemic control were associated with endotoxemia in the present study: branched-chain Aas valine, leucine and isoleucine as well as alanine and histidine were positively associated, whereas glutamine showed negative association with endotoxemia. The branched-chain Aas were previously shown to be associated with insulin resistance and diabetes risk.^31,32^ Mechanistically it was suggested that branched-chain Aas together with lipoprotein-derived lipids affect the responsiveness of peripheral tissues to insulin, as branched-chain Aa-induced insulin resistance in rodents occurs only on the background of a high-fat diet.^29,33^ The high concentrations of triglyceride-rich VLDL particles observed here would be compatible with such a concept.

GlycA derive from glycan *N*-acetylglucosamine residues on enzymatically glycosylated acute-phase proteins, mainly $\alpha_{1}$-acid glycoprotein but also haptoglobin, $\alpha_{1}$-antichymotrypsin, and transferrin.^34^ It is a systemic inflammation marker and a predictor of several incident diseases including T2DM^35^ and CVD.^36^ In our study, LPS was strongly associated with GlycA, and the association was especially prominent in participants suffering from MetS. Both LPS and GlycA have been earlier shown to have a positive association with insulin resistance, serum insulin, triglycerides, total and LDL-cholesterol, and a negative association with insulin sensitivity even in young, overweight women,^37^ emphasizing their role as both mediators and biomarkers of inflammation in metabolic disorders.

There are some limitations concerning our study. Most of the metabolites were highly correlated with each other, which contributed to a substantial number of statistically significant results, despite Bonferroni correction based on PCs. Limulus amebocyte lysate assay, which was used in measuring LPS levels from serum samples, has also drawn some criticism.^38^ In addition, as the study is cross-sectional in nature, no conclusions regarding causal relationships between LPS and metabolite levels can be drawn. The fasting time before sampling differed between the cohorts. In the largest cohort, the FINRISK97, however, the fasting time had only a weak correlation with LPS activity.^5^ The strengths of our study include a relatively large sample size and diverse cohorts representing the whole population from young, healthy adults to the elderly with cardiac problems. Furthermore, our results were highly consistent in the three cohorts.

We showed that endotoxemia associated with lipoprotein profile reflecting many inflammatory features and risk factors for CVD: high VLDL concentration with large particle size and unaltered HDL concentration with small particle size. According to our study, endotoxemia was also associated with saturated FAs, which can act as inflammatory triggers, and GlycA, which are inflammation markers and predictors of several incident diseases and mortality. In conclusion, our findings indicate a strong association of endotoxemia with an adverse metabolic profile linked to cardiometabolic disorders. Thus, serum LPS activity might provide information on the conceivable metabolic profile and the risk of developing these diseases.

## Supplemental Material

sj-xlsx-1-ini-10.1177_1753425920971702 - Supplemental material for Endotoxemia is associated with an adverse metabolic profile Click here for additional data file. Supplemental material, sj-xlsx-1-ini-10.1177_1753425920971702 for Endotoxemia is associated with an adverse metabolic profile by Anne-Mari Määttä, Aino Salminen, Milla Pietiäinen, Jaakko Leskelä, Teemu Palviainen, Wolfgang Sattler, Juha Sinisalo, Veikko Salomaa, Jaakko Kaprio and Pirkko J Pussinen in Innate Immunity

sj-pdf-2-ini-10.1177_1753425920971702 - Supplemental material for Endotoxemia is associated with an adverse metabolic profile Click here for additional data file. Supplemental material, sj-pdf-2-ini-10.1177_1753425920971702 for Endotoxemia is associated with an adverse metabolic profile by Anne-Mari Määttä, Aino Salminen, Milla Pietiäinen, Jaakko Leskelä, Teemu Palviainen, Wolfgang Sattler, Juha Sinisalo, Veikko Salomaa, Jaakko Kaprio and Pirkko J Pussinen in Innate Immunity

sj-xlsx-3-ini-10.1177_1753425920971702 - Supplemental material for Endotoxemia is associated with an adverse metabolic profile Click here for additional data file. Supplemental material, sj-xlsx-3-ini-10.1177_1753425920971702 for Endotoxemia is associated with an adverse metabolic profile by Anne-Mari Määttä, Aino Salminen, Milla Pietiäinen, Jaakko Leskelä, Teemu Palviainen, Wolfgang Sattler, Juha Sinisalo, Veikko Salomaa, Jaakko Kaprio and Pirkko J Pussinen in Innate Immunity

sj-xlsx-4-ini-10.1177_1753425920971702 - Supplemental material for Endotoxemia is associated with an adverse metabolic profile Click here for additional data file. Supplemental material, sj-xlsx-4-ini-10.1177_1753425920971702 for Endotoxemia is associated with an adverse metabolic profile by Anne-Mari Määttä, Aino Salminen, Milla Pietiäinen, Jaakko Leskelä, Teemu Palviainen, Wolfgang Sattler, Juha Sinisalo, Veikko Salomaa, Jaakko Kaprio and Pirkko J Pussinen in Innate Immunity
