# Supplementary material for: Endotoxemia is associated with an adverse metabolic profile
Source: Innate Immun. 2020 Nov 27;27(1):3–14. doi: 10.1177/1753425920971702 (PMC7780360; doi:10.1177/1753425920971702)
Supplement: sj-pdf-2-ini-10.1177_1753425920971702 - Supplemental material for Endotoxemia is associated with an adverse metabolic profile [file sj-pdf-2-ini-10.1177_1753425920971702.pdf]

Supplemental material

Endotoxemia associates with an adverse metabolic profile

Anne-Mari Määttä[1*], Aino Salminen[1], Milla Pietiäinen[1], Jaakko Leskelä[1], Teemu Palviainen[2], Wolfgang Sattler[3], Juha Sinisalo[4], Veikko Salomaa[5], Jaakko Kaprio[2, 6] and Pirkko J. Pussinen[1]

[1]Oral and Maxillofacial Diseases, University of Helsinki and Helsinki University Hospital, Helsinki, Finland
[2]Institute for Molecular Medicine Finland (FIMM), University of Helsinki, Helsinki, Finland
[3] Division of Molecular Biology and Biochemistry, Gottfried Schatz Research Center, Medical University of Graz, Graz, Austria
[4]Department of Cardiology, Heart and Lung Center, Helsinki University Hospital and Helsinki University, Helsinki, Finland
[5]Department of Public Health Solutions, National Institute for Health and Welfare, Helsinki, Finland
[6]Department of Public Health, University of Helsinki, Helsinki, Finland

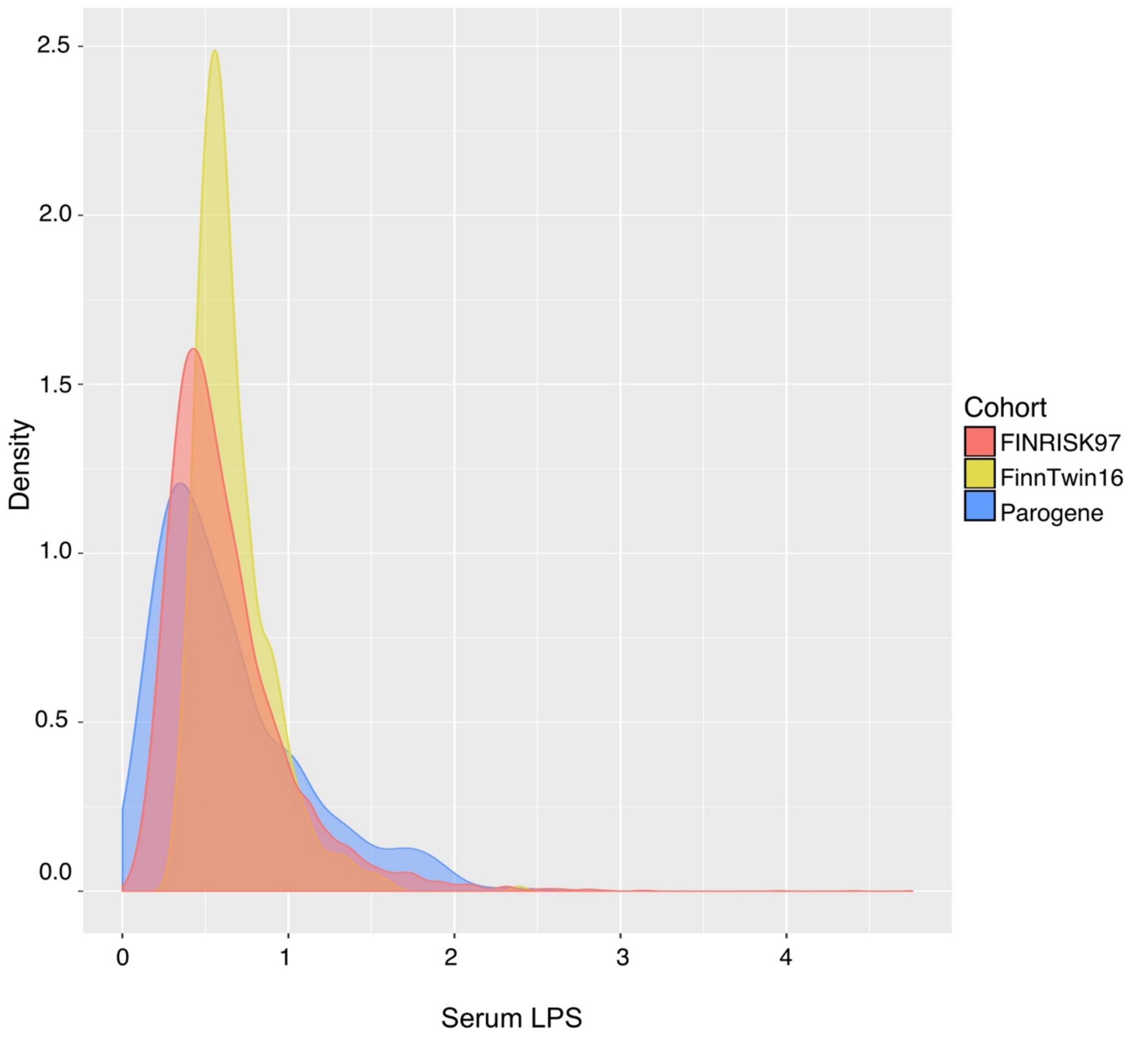

Supplementary Figure 1. LPS distribution in each cohort, density plot. LPS was measured in endotoxin units per millilitre (EU/ml).

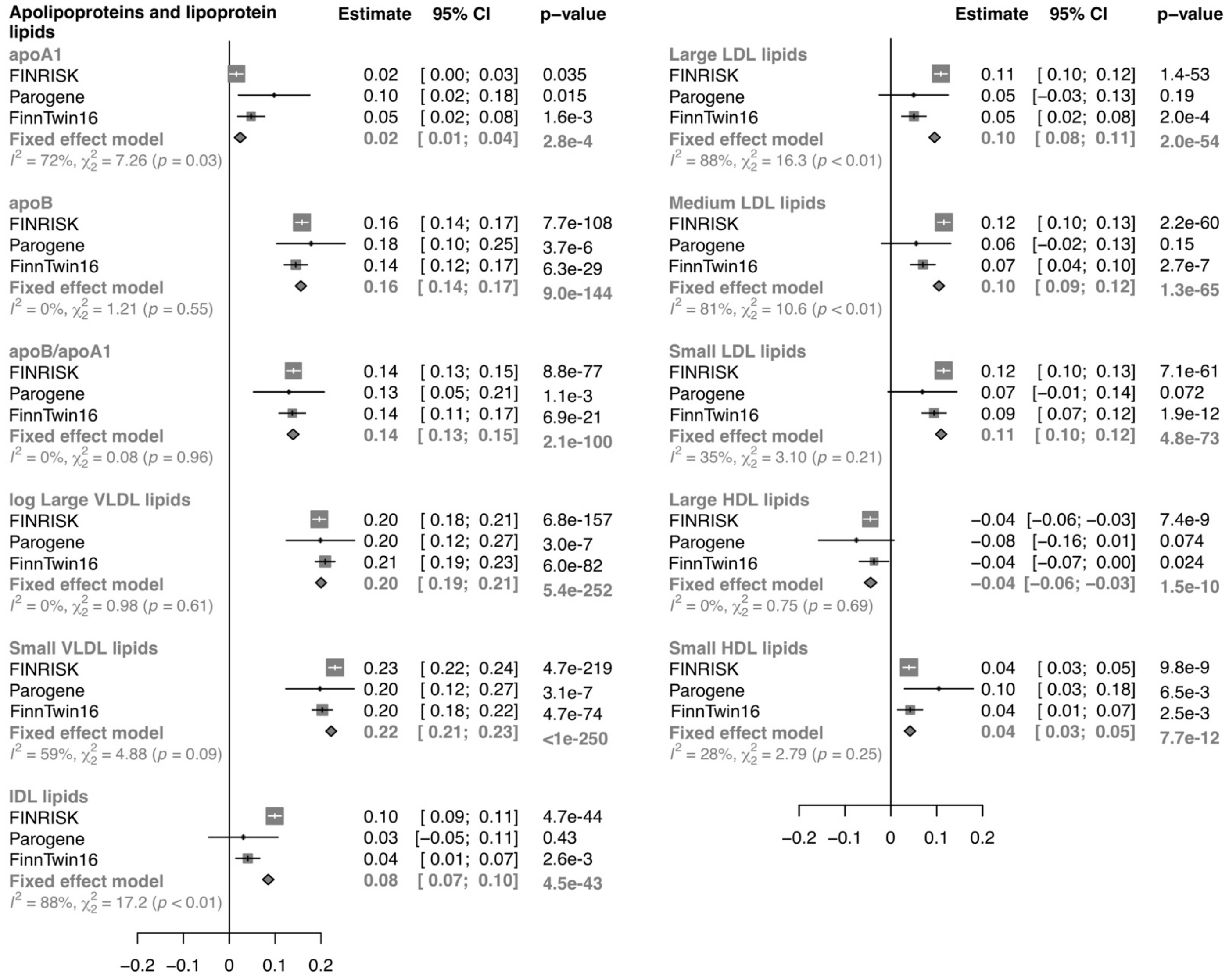

Supplementary Figure 2. The associations of endotoxemia with apolipoproteins and total lipids in lipoprotein subclasses. The β-values and 95% confidence intervals from the analyses of the individual cohorts and from inverse-variance weighted fixed-effect meta-analysis are presented. The linear regression models were adjusted for age, sex, BMI, and current smoking status, as well as kinship in FinnTwin16. Metabolites labeled with “log” were log-transformed to obtain normal distributions. ApoA1 = apolipoprotein A-I, apoB = apolipoprotein B, apoB/apoA1 = ratio of apoB to apoA1.

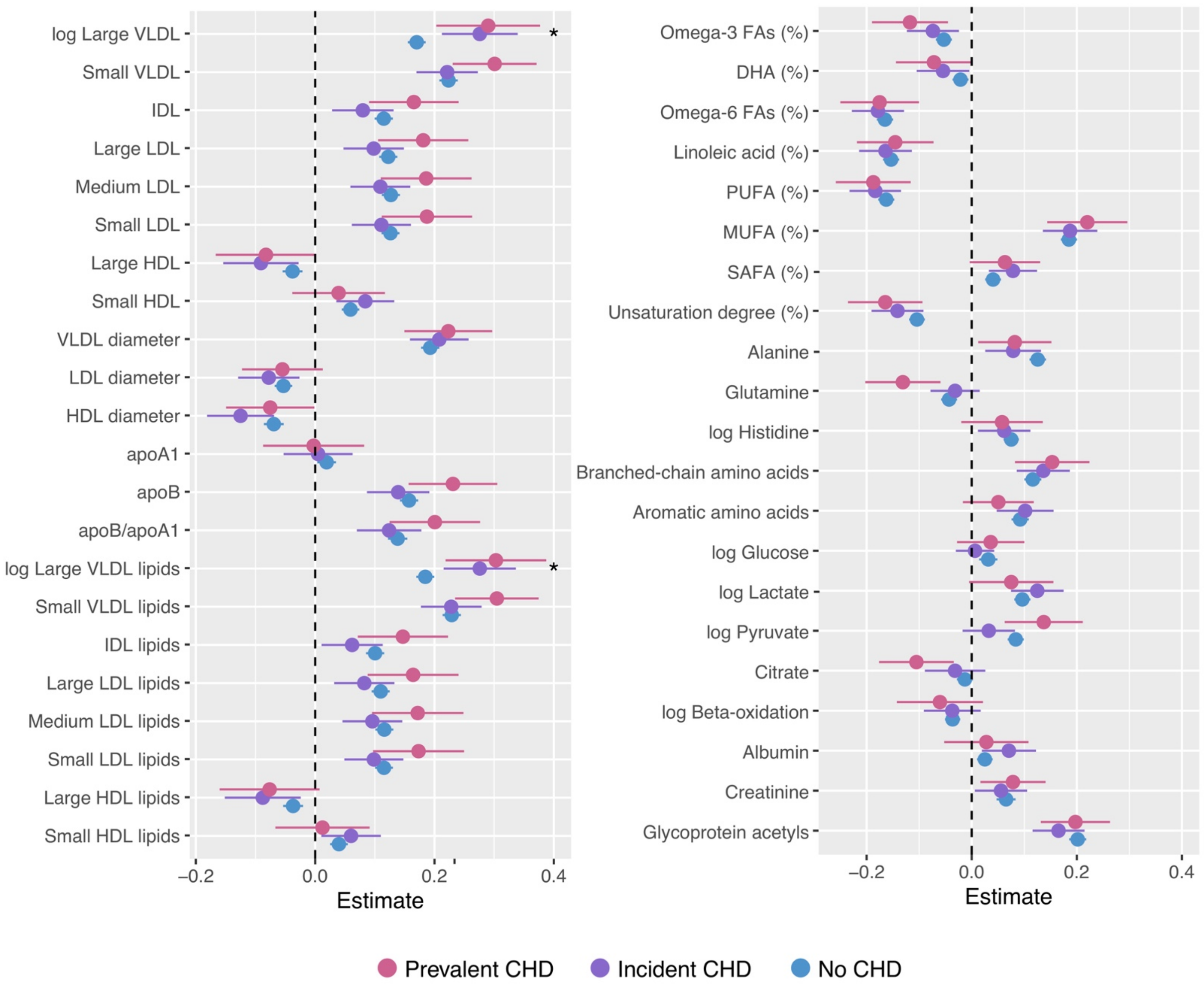

Supplementary Figure 3. The associations between LPS and metabolites in patients with incident or prevalent coronary heart disease (CHD) events and subjects without CHD in FINRISK97. The subjects of FINRISK97 were grouped according to the CHD status. Incident CHD events were reported during the 13-year follow-up. We excluded subjects with prevalent or incident strokes from the analyses. General linear regression models adjusted for age, sex, BMI, and current smoking status were calculated between LPS and the metabolites in different groups. The significance of the differences in the regressions between the groups was analyzed by adding an interaction term of the CHD status with metabolite concentration in the main model and conducting ANOVA. The β-values and 95% confidence intervals are presented. Metabolites labeled with “log” were log-transformed to obtain normal distributions. *: $P < 0.0023$ (ANOVA).

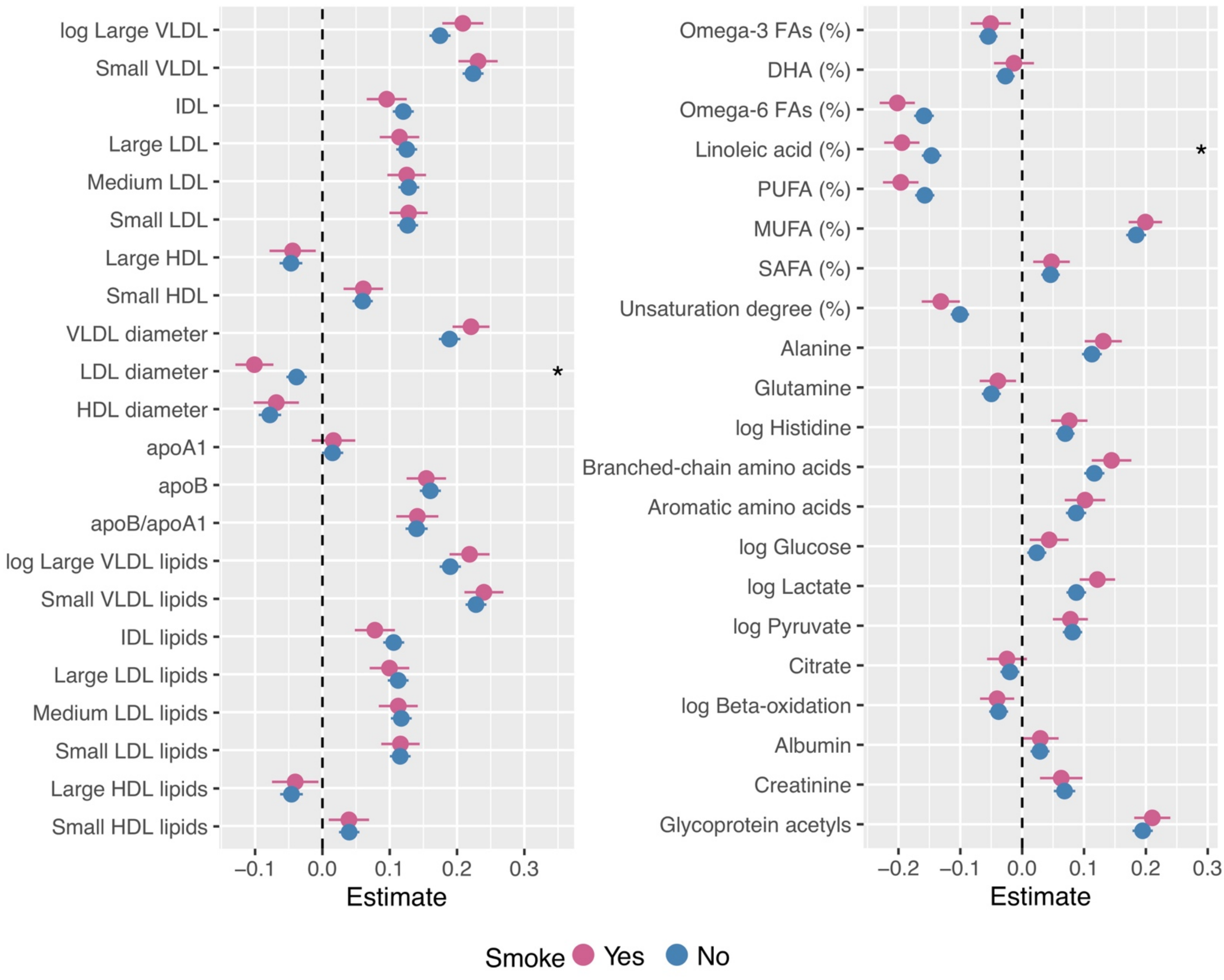

Supplementary Figure 4. The associations between LPS and metabolites in smokers and non-smokers in FINRISK97. The subjects of FINRISK97 were grouped according to the current smoking status. General linear regression models adjusted for age, sex, and BMI were calculated between LPS and the metabolites in different groups. The significance of the differences in the regressions between the groups was analyzed by adding an interaction term of the smoking status with metabolite concentration in the main model and conducting ANOVA. The β-values and 95% confidence intervals are presented. Metabolites labeled with "log" were log-transformed to obtain normal distributions. *: $P < 0.0023$ (ANOVA).

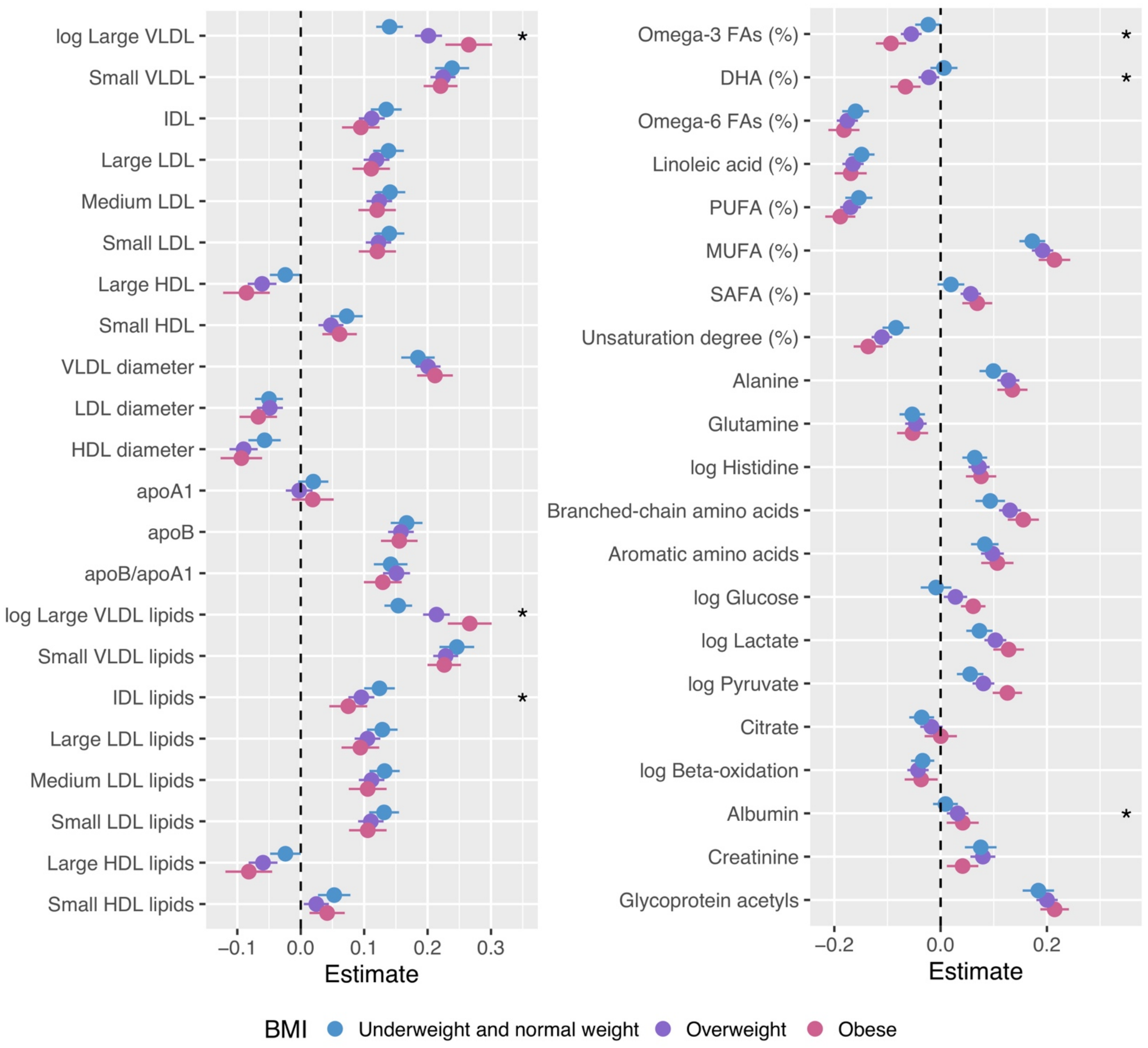

Supplementary Figure 5. The associations between LPS and metabolites in different BMI groups in FINRISK97. The subjects of FINRISK97 were grouped according to BMI into underweight and normal weight (< 25.00), overweight (25.00-29.99), and obese (> 30.0 kg/m^2). General linear regression models adjusted for age, sex, and current smoking status were calculated between LPS and the metabolites in different groups. The significance of the differences in the regressions between the groups was analyzed by adding an interaction term of the BMI group with metabolite concentration in the main model and conducting ANOVA. The β-values and 95% confidence intervals are presented. Metabolites labeled with "log" were log-transformed to obtain normal distributions. *: $P < 0.0023$ (ANOVA).

Supplementary Table 4. C-statistics of the metabolite models for MetS with and without LPS.

	AUC (95% CI)			***P* Value**[1]
	LPS	Metabolite	Metabolite + LPS	
LPS	0.686 (0.671-0.701)			
PUFA (%)		0.766 (0.752-0.779)	0.783 (0.770-0.795)	1.21E-09
MUFA (%)		0.784 (0.771-0.797)	0.795 (0.783-0.808)	2.35E-06
SAFA (%)		0.610 (0.594-0.626)	0.701 (0.686-0.716)	1.03E-30
Unsaturation degree (%)		0.685 (0.670-0.700)	0.733 (0.719-0.747)	8.62E-20
GlycA		0.814 (0.803-0.826)	0.819 (0.807-0.831)	0.00948

[1] *P* Value calculated with paired t-test comparing the AUCs of the metabolite and metabolite + LPS. All models were adjusted for age, sex, smoking, alcohol consumption, and years of education.
